# Detection of two non-synonymous SNPs in *SLC45A2* on BTA20 as candidate causal mutations for oculocutaneous albinism in Braunvieh cattle

**DOI:** 10.1186/s12711-017-0349-7

**Published:** 2017-10-05

**Authors:** Sophie Rothammer, Elisabeth Kunz, Doris Seichter, Stefan Krebs, Martina Wassertheurer, Ruedi Fries, Gottfried Brem, Ivica Medugorac

**Affiliations:** 1 Chair of Animal Genetics and Husbandry, LMU Munich, Veterinaerstr. 13, 80539 Munich, Germany; 2 Tierzuchtforschung e.V. München, Senator-Gerauer-Strasse 23a, 85586 Poing, Germany; 3 Laboratory for Functional Genome Analysis, Gene Center, LMU Munich, Feodor-Lynen-Strasse 25, 81377 Munich, Germany; 4 Institute of Animal Breeding and Genetics, University of Veterinary Medicine Vienna, Veterinaerplatz 1, 1210 Vienna, Austria; 5 Chair of Animal Breeding, TU Munich, Liesel-Beckmann-Strasse (Hochfeldweg) 1, 85354 Freising-Weihenstephan, Germany

## Abstract

**Background:**

Cases of albinism have been reported in several species including cattle. So far, research has identified many genes that are involved in this eye-catching phenotype. Thus, when two paternal Braunvieh half-sibs with oculocutaneous albinism were detected on a private farm, we were interested in knowing whether their phenotype was caused by an already known gene/mutation.

**Results:**

Analysis of genotyping data (50K) of the two albino individuals, their mothers and five other relatives identified a 47.61-Mb candidate haplotype on *Bos taurus* chromosome BTA20. Subsequent comparisons of the sequence of this haplotype with sequence data from four Braunvieh sires and the Aurochs genome identified two possible candidate causal mutations at positions 39,829,806 bp (G/A; R45Q) and 39,864,148 bp (C/T; T444I) that were absent in 1682 animals from various bovine breeds included in the 1000 bull genomes project. Both polymorphisms represent coding variants in the *SLC45A2* gene, for which the human equivalent harbors numerous variants associated with oculocutaneous albinism type 4. We demonstrate an association of R45Q and T444I with the albino phenotype by targeted genotyping.

**Conclusions:**

Although the candidate gene *SLC45A2* is known to be involved in albinism in different species, to date in cattle only mutations in the *TYR* and *MITF* genes were reported to be associated with albinism or albinism-like phenotypes. Thus, our study extends the list of genes that are associated with bovine albinism. However, further research and more samples from related animals are needed to elucidate if only one of these two single nucleotide polymorphisms or the combination of both is the actual causal variant.

**Electronic supplementary material:**

The online version of this article (doi:10.1186/s12711-017-0349-7) contains supplementary material, which is available to authorized users.

## Background

Many cattle breeds are easily recognized by their characteristic coat color patterns that visually separate them from each other, e.g. Pinzgauer with their chestnut base color and a white dorsal and ventral stripe, the white-headed Fleckvieh, or Braunvieh with their eponymous brown color and mealy muzzle. The different colors of hair and eyes in mammals are largely determined by the ratio between eumelanin and pheomelanin, the two main types of melanin [1]. Disruptions in melanin biosynthesis result in a lack or complete absence of pigment in the skin, its appendages and the eyes. These phenotypes are summarized under the term “albinism” and can be broadly assigned to two different categories: oculocutaneous or complete albinism (OCA), which is characterized by complete absence of melanin, and ocular albinism, for which the lack of pigment is only restricted to the eyes [2]. Other disorders that lead to completely or partially white skin and hair are summarized under the term “leucism”. In contrast to albinism, individuals affected by leucism show pigmented irides that can be heterochromatic or vivid blue [3]. Only recently, a study described the case of a single Holstein calf affected by microphthalmia and absence of pigmentation with a phenotype that was most likely caused by an impaired function of the *MITF* gene, which is known to affect pigmentation in various species [4]. Cases of complete or partial albinism have been reported in several cattle breeds and crossbreeds, e.g. Holstein [5–8], Schwarzbuntes Niederungsrind [9], Hereford [10], Guernsey [11], Beef Shorthorn [12], Charolais [13], Braunvieh/Brune des Alpes [14–16] and Fleckvieh [17]. While, in the past, some breeders who were fascinated by the extraordinary phenotype of albino individuals tried to establish pure white populations [5], nowadays albinism is widely regarded as a deleterious trait since affected animals do not fit their breeds’ standards and are more prone to UV damage due to the lack of protective melanin [18]. From a genetic point of view, the mode of inheritance is autosomal recessive in most cases [7, 9, 12, 14, 19]. In Hereford [10, 20] and Fleckvieh with partial albinism [17], dominant inheritance was suggested (OMIA 000204-9913). Since melanogenesis involves numerous proteins and biochemical reaction steps, albinism cannot be traced back to a single gene only [21]. Schmutz et al. [16] identified a frameshift mutation in the *TYR* gene on *Bos taurus* chromosome (BTA) 29 (OMIA 000202-9913) that causes albinism in Brown Swiss cattle, while a premature stop codon in *TYR* (OMIA 000202-89462) is responsible for the same condition in water buffalo [19]. Philipp et al. [17] reported a missense mutation in the *MITF* gene on BTA22 that is accompanied by a white phenotype, heterochromia irides and bilateral hearing loss in German White Fleckvieh.

In this paper, we present a case of oculocutaneous albinism in two related Braunvieh calves that is most probably caused by a mutation in the *SLC45A2* gene on BTA20.

## Methods

### Animal samples

Two Braunvieh calves (BV-ALBINO1 and BV-ALBINO2) with oculocutaneous albinism were reported on a private farm. Both animals were fathered by the same sire and displayed a phenotype that was characterized by a complete lack of pigment in the skin, hair, eyes, horns and hooves. The albino phenotype was not present in either of their parents, which were half-sibs. Moreover, besides these two albino calves, the bull had also sired 14 calves with a wild type color pattern. For these reasons, we hypothesized that this trait had a recessive mode of inheritance.

Blood and hair samples from both calves, their dams and eight other relatives were collected on the farm. Since the bull was used as a natural service sire and had already been slaughtered before the birth of the albino calves, we did not have access to tissue samples of their sire. Samples of the other animals used in this study were either already stored at the LMU Chair of Animal Genetics and Husbandry and originated from previous research projects or were stored at the Tierzuchtforschung e.V. München after paternity control. Table 1 summarizes information on the breeds and numbers of animals included in this study and on the methods applied.

**Table 1:** List of breeds and number of animals included in the analysis and methods applied

Breed or group	Total	KASP™	RFLP	Mapping	WGS
Albino-Braunvieh	2	2	2	2	1^b^
Albino-Braunvieh relatives^a^	10	10	10	7	–
Braunvieh	327	263	281	51	4^c^
Original Braunvieh	38	23	24	34	–
Croatian Busha	6	6	6	–	–
BV-crossbred	2	2	2	–	–
Galloway	10	10	10	–	–
Lakenvelder	12	12	12	–	–
Diverse breeds (1000 bull genomes project)	–	–	–	–	1682
Aurochs [26]	–	–	–	–	1
Total	407	328	347	94	1688

Number of animals per breed group, which were used either for mapping candidate haplotypes and/or genotyping of candidate variants by KASP™ (T444I) and/or RFLP (R45Q)

The last two columns show the numbers of animals, which were used for the initial haplotype-based mapping procedure and for whole-genome sequencing (WGS) analyses

^a^Two dams and eight close relatives of the two albino calves sampled on the same farm

^b^Sequenced in this study

^c^Sequenced in a previous study [29] and used for variant calling with *SAMtools mpileup*

### Genotyping

In this study, samples from both albino calves, their mothers, and one other relative were genotyped, while four related artificial insemination bulls and 85 unrelated animals had already been genotyped in previous studies using the Illumina Bovine SNP50 BeadChip (Illumina, San Diego, USA). Chromosomal positions of all single nucleotide polymorphisms (SNPs) were determined according to release UMD 3.1 of the *Bos taurus* reference genome [22]. In a subsequent filtering step, we excluded SNPs (1) that were successfully genotyped in less than 95% of the animals, (2) that frequently showed paternity conflicts in animals with known ancestry, and (3) for which physical positions in the reference genome were unknown or ambiguous. Reconstruction of haplotypes and imputation of missing genotypes were performed with the program BEAGLE 3.0.4 [23], which operates on a hidden Markov model.

### Identification of a common haplotype

In order to identify a common haplotype associated with the cases of oculocutaneous albinism in this study and, thus, to narrow down the chromosomal intervals that may contain the causative mutation(s), SNP genotypes of the two albino animals, their relatives and a representative sample of the Braunvieh and Original Braunvieh breeds (Table 1) were analyzed using sliding windows with a fixed size of 40 SNPs. Under the assumption of a recessive mode of inheritance, only haplotypes that were homozygous in both albino individuals, heterozygous in their dams, and heterozygous or not present in all other animals were considered for further analyses.

### Generation of sequence data and identification of candidate causal mutations

DNA of the albino animal BV-ALBINO1 was sequenced at the Gene Center of the LMU Munich. A sequencing library was prepared from 1 µg of genomic DNA with the 1S kit from Swift Biosciences (Ann Arbor, USA) and sequenced on one lane in 2*100 bp mode on a HiSeq 1500 instrument (Illumina, San Diego, USA). Sequence reads were then aligned to the UMD 3.1 reference sequence using the Burrows-Wheeler Aligner (BWA) [24]. Variant calling was performed with SAMtools mpileup [25] including sequence data from four additional unrelated Brown Swiss animals and the Aurochs genome [26]. The Ensembl variant effect predictor [27] was used to annotate coding variants within the interval 24,179,172–71,793,734 bp on BTA20 based on Ensembl Release 85 and RefSeq Release 76. The genotypes of candidate causal variants were validated in 1682 animals from various bovine breeds that were available from the fifth run of the 1000 bull genomes project [28] and in an in-house genotype database [29].

### Targeted genotyping by PCR–RFLP and KASP™

In order to exclude or verify the SNP at position 39,829,806 bp on BTA20 as a candidate causal mutation, targeted genotyping by PCR–RFLP was carried out on an extended sample set (Table 1) including additional animals and breeds. After amplification of the DNA segment of interest using the primers 5′-GCTCCATGTCAAATCCACCT-3′ and 5′-CCCAGCCTACCTAGCCTACC-3′ and digestion with the restriction enzyme *Msp*I, the resulting fragments were separated and visualized by 2% ethidium bromide-stained agarose gel electrophoresis. For the second SNP at position 39,864,148 bp on BTA20, targeted genotyping was carried out on the same sample set with a specifically designed KASP™ assay (LGC, Teddington, UK).

## Results

### Results of the pedigree analysis

As shown in Additional file 1: Fig. S1, both albino individuals were produced by half-sib mating. It is also clear that the bull B1, who was the sire of the albinos’ sire S1 and thus their grandsire, carried the albino candidate mutations since these were transmitted to at least four of his descendants. We were also able to show that the sire of bull B1 did not carry these mutations. However, since the dam and granddam of B1 are no longer alive and the grandsire is unknown, it remains unclear if B1 inherited the mutations from his dam or if they occurred spontaneously. Nevertheless, analysis of the pedigree strongly supports a recessive mode of inheritance.

### Results of the mapping and genotyping analyses

**Fig. 1:**
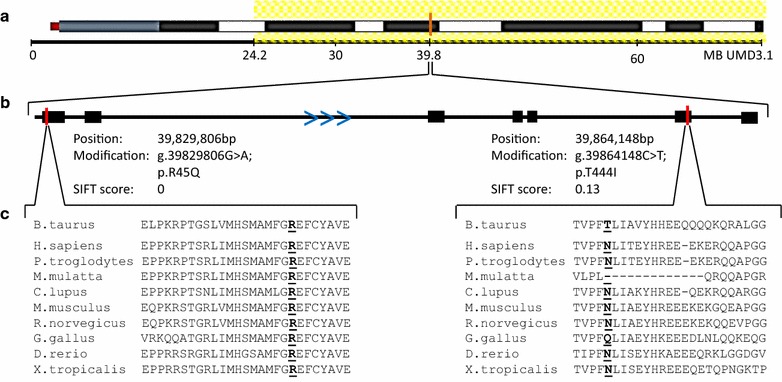
**a** Schematic overview of BTA20. Yellow shaded parts mark the candidate haplotype, an orange line indicates the position of *SLC45A2* [chromosome adapted to the illustration on CattleQTLdb (http://www.animalgenome.org)]. **b** Schematic overview of the *SLC45A2* gene sequence. Blue arrows indicate the orientation of transcription of SLC45A2. Red lines mark the positions of the two candidate mutations. Exact position according to UMD 3.1, modification and SIFT score are given for each SNP. **c** Excerpt of the multi-species alignment performed by HomoloGene that clearly shows that p.R45Q is conserved in contrast to p.T444I (indicated in bold face)

Analysis of genotyping data for the two albino calves, their dams, five relatives and 85 unrelated Braunvieh and Original Braunvieh animals resulted in the detection of a 47.61-Mb haplotype between SNP positions 24,179,723 and 71,793,734 bp on BTA20 (Fig. 1a), which was exclusively homozygous in both albino individuals, heterozygous in their dams, and heterozygous or absent in any of the other animals. Besides this extended haplotype, five shorter haplotypes also met these criteria (Table 2). Comparisons of the SNP positions with a list of genes (Table 3) that are known to be involved in albinism showed that *SLC45A2* (*solute carrier family 45 member 2*) on BTA20: 39,829,673–39,867,694 bp was located within the borders of the largest stretch of extended homozygosity. Sequence comparisons between animal BV-ALBINO1, four Braunvieh sires and the Aurochs genome led to the detection of two private alleles at positions 39,829,806 bp (G/A; R45Q) and 39,864,148 bp (C/T; T444I). Both of these SNPs (see Additional file 2: Fig. S2 and Additional file 3: Fig. S3), which were not present in 1682 animals from the 1000 bull genomes project, are located within the *SLC45A2* gene.

**Table 2:** Candidate haplotypes

Chr	Start	End	Mb	Number of SNPs
8	83,888,935	86,613,020	2.72	57
20	24,179,723	71,793,734	47.61	981
27	12,330,184	16,012,903	3.68	89
27	24,144,679	25,776,811	1.63	40
27	25,879,452	31,426,001	5.55	103
27	32,671,451	45,368,987	12.70	262

Haplotypes that were homozygous in both albino calves, heterozygous in their dams, and not present in a homozygous state in any of the other animals

**Table 3:** Albinism genes

Type of albinism	Locus	Position (UMD 3.1, ENSEMBL)
Oculocutaneous albinism type 1	*TYR*	BTA29: 6,351,877–6,462,240
Oculocutaneous albinism type 2	*OCA2*	BTA2: 347,042–598,104
Oculocutaneous albinism type 3	*TYRP1*	BTA8: 31,710,698–31,726,956
Oculocutaneous albinism type 4	*SLC45A2*	*BTA20: 39,829,673*–*39,867,694*
Hermansky-Pudlak Syndrome type 1	*HPS1*	BTA26: 19,361,232–19,386,569
Hermansky-Pudlak Syndrome type 2	*AP3B1*	BTA10: 9,040,253–9,300,567
Hermansky-Pudlak Syndrome type 3	*HPS3*	BTA1: 119,944,594–119,985,509
Hermansky-Pudlak Syndrome type 4	*HPS4*	BTA17: 68,368,661–68,396,020
Chediak-Higashi Syndrome	*LYST*	BTA28: 8,423,715–8,567,655
Ocular albinism type 1	*GPR143*	BTAX: 143,861,798–143,891,357

Genes causing albinism according to the albinism database provided by the University of Minnesota (http://www.ifpcs.org/albinism/) and their positions in the cattle genome. The position highlighted in italics was included in one of the candidate haplotypes given in Table 2

Targeted genotyping by PCR–RFLP and the KASP™ assay confirmed both SNPs at positions 39,829,806 and 39,864,148 bp on BTA20 as candidate causal mutations. Both albino animals were homozygous for the alternative alleles, whereas their dams and four other relatives, which could be traced back to the same ancestor bull, showed heterozygous genotypes. All other animals were homozygous for the respective reference alleles. However, SIFT scores, which were calculated with the Ensembl variant effect predictor [27] (damaging variant when the score is equal to 0.05 or less, tolerated variant when the score is between 0.05 and 1), classified the R45Q mutation as damaging (SIFT score 0), whereas the T444I mutation was predicted as tolerable (SIFT score 0.13, Fig. 1b). Furthermore, protein sequence comparisons between *Bos taurus* and several other species showed that the arginine residue (R45Q) is highly conserved throughout eukaryotes and even more distant species, whereas the threonine residue (T444I) is not (Fig. 1c).

## Discussion

Analysis of sequence data from a Braunvieh animal with oculocutaneous albinism revealed two non-synonymous SNPs in the *SLC45A2* gene on BTA20 that were both confirmed as candidate causal mutations by targeted genotyping. Since the mapping procedure was based on simple assumptions (recessive inheritance, mutation not in a common haplotype) and since, in general, the study was based on a candidate gene approach, we could not irrevocably prove that no other equally fitting candidate mutation(s) exist. However, the distribution of the genotypes of g.39829806A>G and g.39864148C>T as well as the affected gene *SLC45A2* strongly suggest a causal connection between the albino phenotype of the two Braunvieh calves and *SLC45A2*. Mutations in *SLC45A2* (also known as *membrane*-*associated transporter protein*, *MATP*, or *antigen isolated from immunoselected melanoma 1*, *AIM1* [30]) are known to cause a wide range of phenotypes that are characterized by reduced levels or complete absence of melanin synthesis in a variety of species, e.g. white tigers [31], cream-colored horses [32], hypopigmented medaka fish [33], mice with the underwhite (uw) phenotype [34, 35], and chicken and Japanese quail with silver or cinnamon plumage color or imperfect albinism [36]. Furthermore, OCA in a Western lowland gorilla [37], white Doberman pinscher dogs [38] and several long-haired dog breeds [39] was also traced back to mutations in *SLC45A2*. In zebrafish, Irion et al. [40] demonstrated that repair of a premature stop codon in the *SLC45A2* gene using CRISPR/Cas9 led to the production of melanin pigment in the formerly albinotic fish, thus confirming the findings of Dooley et al. [41] who had identified an association between this gene and the albino phenotype. In humans, it has been shown that mutations in *SLC45A2* are not only the cause of OCA4 [34, 42–47], but that they also play a role in normal skin color variation [48, 49].

At the cellular level, Costin et al. [50] suggested that *SLC45A2* is involved in the processing and intracellular transport of tyrosinase and other proteins in the melanosome. Due to structural and sequence similarities with plant sucrose transporters, it was also speculated that SLC45A2 might be a transporter for substrates of melanin biosynthesis in the melanosomal membrane [33] or, if it co-transported protons, it might regulate melanosome acidification and thus its function [34]. Dooley et al. [41] showed that SLC45A2 does indeed functions as a Na^+^/H^+^ exchanger that is involved in maintaining pH and ion homeostasis in the melanosome, which in turn influences tyrosinase activity, possibly by copper binding [51]. The transcription of *SLC4A2* itself is regulated by the transcription factor MITF [35].

**Fig. 2:**
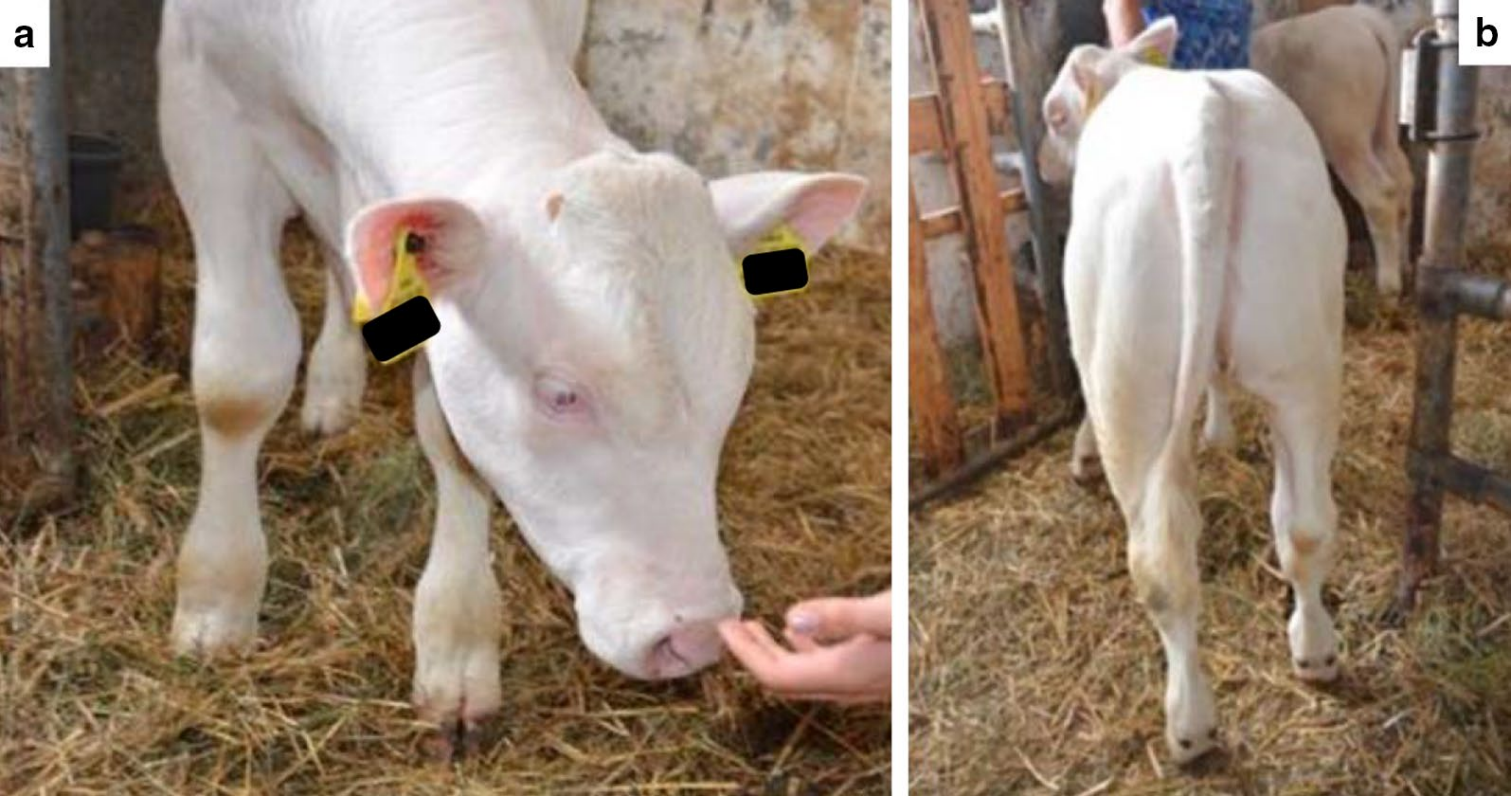
Pictures of the Braunvieh albino calves. **a** Illustration of the absence of pigment in the eyes. **b** Illustration of the unusual muscling of the calves

In summary, OCA in the two Braunvieh calves analyzed in our study is most possibly caused by a base change in the *SLC45A2* gene. However, the question of which of the two non-synonymous SNPs at positions 39,829,806 bp (G/A; R45Q) and 39,864,148 bp (C/T; T444I) is actually the causal mutation, could not be answered yet. Based on the SIFT score results and the fact that a highly conserved arginine residue was identified throughout eukaryotes and more distant species (Fig. 1c), the SNP at position 39,829,806 bp (R45Q) is the most probable causal mutation. Moreover, there also exists the possibility that both mutations are needed to cause the albino phenotype. For example, a similar case of two disease-associated missense mutations that were found on a single haplotype was described for bovine congenital pseudomyotonia [52]. The relatively long common SNP-haplotype (47.61-Mb) that harbored the two candidate mutations, the perfect linkage disequilibrium between both mutations in the investigated samples, and their absence in all Braunvieh animals that were sampled outside of the case herd suggest that both the causal and the hitchhiking mutation were of recent origin, i.e. that they most probably occurred in the case herd recently. Since both mutations are located 34 kb apart, an independent mutation event is hypothesized. Assuming a genetic distance of 0.034 cM (i.e. 1 Mb = 1 cM), one recombination per 2941 gametes is expected. Therefore, our chances of sampling an animal that carries only one mutation are very small. An alternative approach would be to produce additional albino animals by mating live heterozygous animals. The albino offspring could then be used for more precise phenotyping. Both BV-ALBINO1 and BV-ALBINO2 showed an unusual muscularity for the Braunvieh breed (Fig. 2). Unfortunately, both calves were slaughtered before we had the opportunity to phenotype them more precisely. The confirmation of this phenotype in additional animals born in independent environments could be of interest for the investigation of a possible pleiotropic effect of one or both mutations. Possible pleiotropy is, at this stage, highly speculative, but we would like to draw the attention to the fact that according to the EMBL-EBI Expression Atlas [53], a low expression of the *SLC45A2* gene is observed in bovine muscles, too. Analogous to Irion et al. [40], the effects of both mutations could be tested with the help of the CRISPR/Cas9 methodology [54], if a suitable cell line of a model organism was found.

## Conclusions

Two non-synonymous SNPs at positions 39,829,806 bp (G/A; R45Q) and 39,864,148 bp (C/T; T444I) in the *SLC45A2* gene on BTA20 were identified as candidate causal mutations for oculocutaneous albinism in two Braunvieh calves. Thus, *SLC45A2* can be added to the list of genes that are responsible for OCA in cattle. To determine which of the two mutations or even if the combination of both is causal for oculocutaneous albinism in Braunvieh, it would be necessary to produce additional cases and investigate them more precisely.

## Additional files

**Additional file 1: Fig. S1.** Pedigree of the albino calves. As the red highlighted paths show, both albinos and all carriers of the SNPs g.39829806G>A and g.39864148C>T can be traced back to a single natural service sire (B1). Symbols are as follows: squares = males, circles = females, filled symbols = albinos (homozygous for both SNPs), symbols with dot inside = heterozygous individuals, symbols with question mark inside = genotype unknown, crossed out symbols = no material available. For bull S2, 50K genotype data was available from previous studies; however, no material for targeted genotyping was available.

**Additional file 2: Fig. S2.** IGV screenshot showing the candidate SNP on BTA20 at position 39,829,806 bp (g.39829806G>A, p.R45Q).

**Additional file 3: Fig. S3.** IGV screenshot showing the candidate SNP on BTA20 at position 39,864,148 bp (g.39864148C>T, p.T444I).
