# Supplementary figures and images for: Detection of two non-synonymous SNPs in SLC45A2 on BTA20 as candidate causal mutations for oculocutaneous albinism in Braunvieh cattle
Source: Genet Sel Evol. 2017 Oct 5;49:73. doi: 10.1186/s12711-017-0349-7 (PMC5628493; doi:10.1186/s12711-017-0349-7)

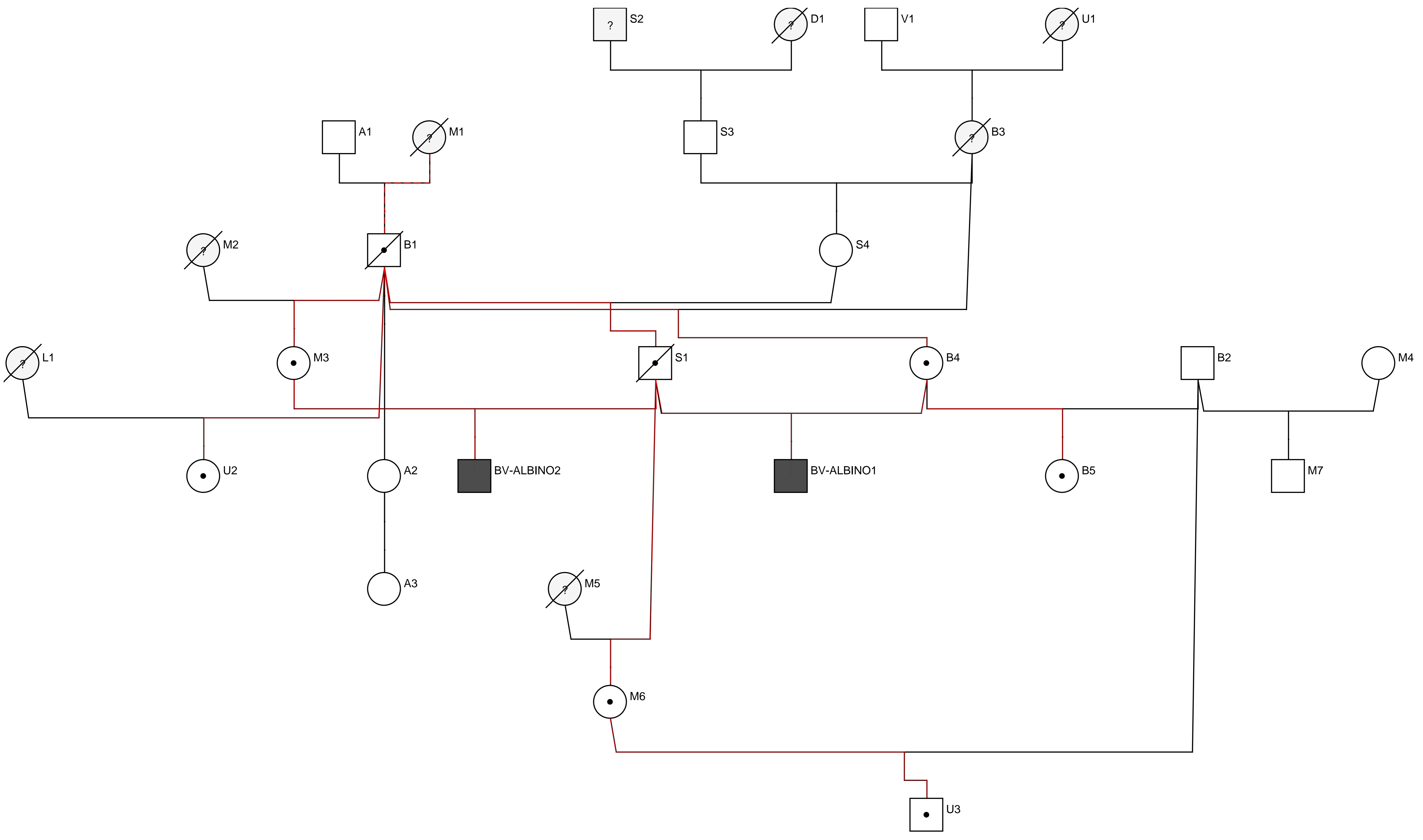

Supplement: Supplementary file 1 — Additional file 1: Fig. S1. Pedigree of the albino calves. As the red highlighted paths show, both albinos and all carriers of the SNPs g.39829806G>A and g.39864148C>T can be traced back to a single natural service sire (B1). Symbols are as follows: squares = males, circles = females, filled symbols = albinos (homozygous for both SNPs), symbols with dot inside = heterozygous individuals, symbols with question mark inside = genotype unknown, crossed out symbols = no material available. For bull S2, 50K genotype data was available from previous studies; however, no material for targeted genotyping was available. [file 12711_2017_349_MOESM1_ESM.pdf]

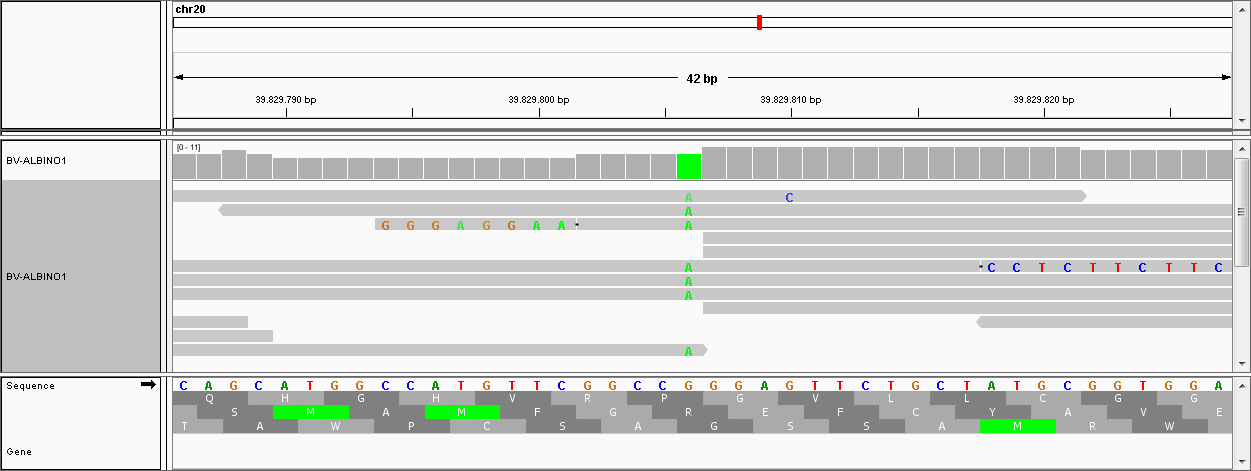

Supplement: Supplementary file 2 — Additional file 2: Fig. S2. IGV screenshot showing the candidate SNP on BTA20 at position 39,829,806 bp (g.39829806G>A, p.R45Q). [file 12711_2017_349_MOESM2_ESM.png]

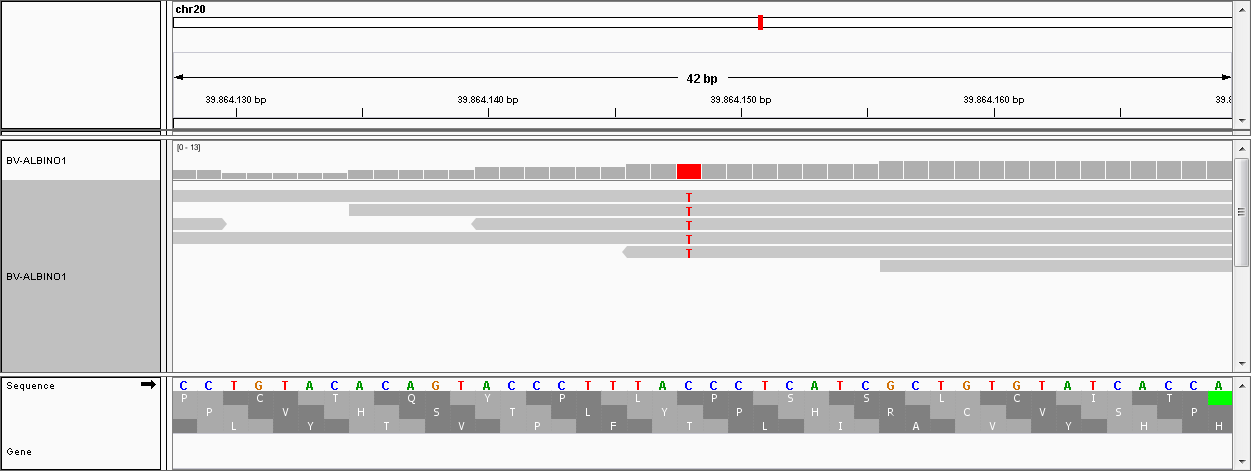

Supplement: Supplementary file 3 — Additional file 3: Fig. S3. IGV screenshot showing the candidate SNP on BTA20 at position 39,864,148 bp (g.39864148C>T, p.T444I). [file 12711_2017_349_MOESM3_ESM.png]
